# 난임 여성의 난임관련 삶의 질 영향요인

**DOI:** 10.4069/kjwhn.2020.03.08

**Published:** 2020-03-19

**Authors:** Yun Mi Kim, Ju-Hee Nho

**Affiliations:** College of Nursing, Jeonbuk Research Institute of Nursing Science, Jeonbuk National University, Jeonju, Korea; 전북대학교 간호대학•간호과학연구소

**Keywords:** Infertility, Fatigue, Depression, Marriage relationship, Quality of life, 난임, 피로, 우울, 부부 친밀도, 삶의 질

## Abstract

**Purpose:**

Infertile women experience various physical, psychological, and relational problems that affect their infertility–related quality of life (QoL). This study investigated infertile women's infertility-related QoL with the goal of identifying how it is influenced by fatigue, depression, and marital intimacy.

**Methods:**

A sample of 140 infertile women was surveyed in a cross-sectional study. Data were collected from February to April 2018 using self-report structured questionnaires at three infertility clinics located in Jeonju, Korea. Data were analyzed using the independent t-test, analysis of variance, Pearson correlation coefficients, and stepwise multiple regression analysis in SPSS for Windows version 25.0. The subjects agreed to complete a face-to-face interview, including administration of the Fatigue Severity Scale, Depression Anxiety Stress Scale-21, Marital Intimacy Scale, and the Fertility Quality of Life tool.

**Results:**

The mean age of the participants was 35.6±4.3 years. Infertility-related QoL was negatively correlated with fatigue (r=–.42, *p*<.001) and depression (r=–.56, *p*<.001), and positively correlated with marital intimacy (r=.30, *p*<.001). Multiple regression analysis showed that depression (β=–0.44, *p*<.001), fatigue (β=–0.27, *p*<.001), and husband's attitude (β=–0.19, *p*=.007) had significant effects on the QoL of infertile women, accounting for 40.5% of the variance in infertility-related QoL.

**Conclusion:**

The study provides insights into how infertile women's infertility-related QoL was influenced by depression, fatigue, and their husbands' attitudes regarding infertility treatment. To improve infertile women's infertility-related QoL, healthcare providers should consider developing strategies to decrease depression and fatigue in infertile women and to address their husbands' attitudes.

## Introduction

### 연구 필요성

한국의 여성 평균 초혼 연령은 1990년에 24.8세였던 이후로 2018년 평균 30.4세로 지속적으로 증가하고 있다[1]. 여성의 초혼 연령 증가에 따라 모성의 평균 출산 연령도 함께 증가되어, 2018년 평균 출산 연령은 32.8세였으며, 35세 이상의 고령 산모 출산 비중은 31.8%로 전년 대비 2.4% 증가하였다[1]. 이와 같이 가임기 여성의 연령이 지속적으로 증가함에 따라 감소된 신체적 및 생리적 상태는 생식능력의 감소로 이어져 난임의 과정을 겪게 된다[2]. 난임이란 피임을 하지 않고 정상적인 성생활을 하고 있음에도 1년 이내에 임신이 되지 않는 상태로, 2018년 보고된 보건사회연구원 자료에 따르면 유배우 여성의 난임 진단율은 52.1%로 2015년의 조사 결과(37.1%)와 비교했을 때 15.0% 증가하였다[3]. 난임 여성 비율은 점차적으로 증가하고 있는 상황으로, 난임 여성들은 반복적인 진료와 혈액 검사, 소변 검사, 호르몬 검사, 나팔관 조영술, 배란 검사, 초음파 검사, 배우자의 정자 검사 같은 사전 검사 및 시술 과정을 거치게 된다. 이러한 반복적인 진료과정을 통해서도 임신을 보장받는 것은 아니므로 난임 여성은 신체적, 심리적, 관계적인 측면의 다양한 문제를 겪고 삶의 질에 영향을 받게 된다[4]. 삶의 질에 영향을 주는 요인 중 하나인 신체적 문제들은 난임 여성에게 다양하게 나타나는데 검사와 치료를 위한 투약 등으로 기능적 질환과 비슷한 신체적 증상들이 유발된다[5]. 난임 여성이 자주 경험하는 신체적 증상은 피로, 설사, 변비, 불면증 등이 있고[5], 그 중 피로는 난임 여성이 쉽게 느끼며 자주 표현하는 증상이다. 난임 여성은 정상 임신 과정을 겪었던 여성이나 일반 여성들에 비해 더욱 심한 피로를 경험한다고 하였으나[6], 난임 여성의 피로가 어느 정도이고 삶의 질에 미치는 영향이 어느 정도인지에 대한 연구는 찾기 힘든 실정이다. 따라서 난임 여성의 신체적 증상으로 나타나는 피로의 정도를 파악하고 삶의 질에 대한 영향을 파악하여 난임 여성의 피로를 감소시킬 수 있는 전략이 필요하다. 난임 여성은 우울, 난임 관련 스트레스, 불안, 죄책감 등[7] 다양한 심리적 문제를 경험한다. 이 중 우울은 난임 여성이 경험하는 가장 대표적인 심리적 문제이며[8], 난임 관련 삶의 질에 영향을 주는 주요 원인이다[9]. 난임 치료 과정 중인 여성의 우울을 측정한 연구에서 난임 여성의 우울 정도는 중간 이상으로 확인되었고[10], 보조생식술을 받는 여성의 94.5%가 우울한 상태라고 하였다[8]. 국내 141명의 난임 여성을 대상으로 한 연구에서 우울과 난임 관련 삶의 질은 음의 상관관계가 있으며, 심리적 중재가 필요할 정도의 높은 우울과 낮은 난임 관련 삶의 질을 나타낸다고 보고하였다[11].

결혼과 함께 임신을 위한 과정은 한 개인인 여성을 포함한 부부가 겪는 위기 사건이며 부부의 공동 상황이고 공동 과업이다[10]. 이에 난임을 경험하는 부부는 임신이라는 과업 달성을 향한 어려운 과정에서 부부 친밀도가 낮아지고[12] 이는 난임 관련 삶의 질에 영향을 미치게 된다. 부부 친밀도는 부부가 상호적으로 느끼고 공유하는 밀접함으로 정의된다[13]. 난임 여성의 부부 친밀도를 확인한 체계적 문헌고찰 연구에서는 난임 여성이 임신 여성보다 부부 친밀도가 덜 안정적이라고 하였고[12], 배우자와의 관계가 만족스러울수록 삶의 질이 높다고 하였다[8]. 이와 같이 난임 여성의 피로, 우울과 부부 친밀도는 난임 관련 삶의 질과 관련이 있고, 정상 임신 여성에 비해 심각한 문제를 경험하는 난임 여성[6,12]의 신체, 심리, 관계적인 건강과 삶의 질에 대한 의료진의 관심과 중재가 필요하다.

난임 여성의 삶의 질은 피로, 우울과 부부 친밀도뿐 아니라, 교육수준, 수입, 직업, 거주 지역, 아이를 갖고 싶은 의지, 결혼과 난임 기간 등에 따라서도 다르게 나타났다[14,15]. 또한 국내 125명의 난임 여성을 대상으로 한 연구에서는 직업 유무, 시술비용에 대한 부담감이 난임 관련 삶의 질에 영향을 미치는 것으로 보고되어[9] 다양한 인구학적 요소들이 난임 관련 삶의 질에 영향을 미치는 것을 알 수 있다.

이와 같이 난임 여성의 삶의 질은 임신 여성보다 낮으며, 인구학적 특성을 비롯한 신체적, 심리적, 관계적 요소들이 영향을 주는 것으로 보고되었다. 그러나 대부분의 난임 여성의 삶의 질에 대한 연구는 임신 여성과의 비교 또는 각각 영역의 요소들과 삶의 질과의 관련성을 확인한 연구가 대부분이었다. 즉, 난임 여성과 임신 여성의 삶의 질 비교[11,14], 우울, 불안, 난임 관련 스트레스 등의 심리적 요소와 삶의 질과의 관련성[8,9], 부부 친밀도와 삶의 질 관련성[9,12], 삶의 질의 영역별 비교[7], 인구학적 요소와 삶의 질과의 관련성 연구[14,15] 등이 대부분으로, 난임 여성의 난임 관련 삶의 질 확인과 영향요인을 파악하기 위한 다양한 영역의 요소를 포괄적으로 확인한 연구는 찾기 어려운 실정이다.

이에, 본 연구에서는 난임 여성의 난임 관련 삶의 질을 확인하고 삶의 질의 영향요인으로 인구학적 특성을 비롯한 신체적 측면의 피로, 심리적 측면의 우울, 관계적 측면의 부부 친밀도를 파악하여 난임 관련 삶의 질에 영향을 미치는 요인을 통합적으로 확인해보고자 한다.

### 연구 목적

본 연구는 난임 여성의 인구학적 특성, 피로, 우울, 부부 친밀도와 난임 관련 삶의 질의 정도를 확인하고 삶의 질에 영향을 미치는 요인을 파악하고자 하는 연구이다. 이를 통해 난임 여성의 삶의 질을 향상시킬 수 있는 간호학적인 상담과 중재 프로그램 개발을 위한 기초자료를 마련하고자 한다.

### 본 연구의 구체적 목적은 다음과 같다.

1) 난임 여성의 피로, 우울, 부부 친밀도와 난임 관련 삶의 질 정도를 파악한다.

2) 난임 여성의 일반적 특성에 따른 피로, 우울, 부부 친밀도와 난임 관련 삶의 질 차이를 파악한다.

3) 난임 여성의 피로, 우울, 부부 친밀도와 난임 관련 삶의 질 간의 관계를 파악한다.

4) 난임 여성의 난임 관련 삶의 질에 미치는 영향요인을 파악한다.

## Methods

Ethics statement: This study was approved by the Institutional Review Board of Presbyterian Medical Center (IRB No. 2018-01-002). Informed consent was obtained from the subjects.

### 연구 설계

본 연구는 난임 여성의 난임 관련 삶의 질에 미치는 영향요인을 확인하기 위한 상관성 조사 연구이다.

### 연구대상 및 표집방법

본 연구는 전주에 소재한 난임 전문병원 3곳에서 난임 진단을 받고 의사에게 1회 이상의 상담(자연주기법) 및 시술(배란 유도, 인공수정, 체외수정)을 받는 여성을 대상으로 편의 추출하였다. 본 연구의 표본크기는 G*power program 3.1.9.2을 이용하여 산출하였다. 연구 분석에 사용될 통계적 검정법에 따라, 회귀분석에 필요한 적정 표본크기는 유의수준 .05, 일반적 특성(나이, 교육, 배우자와의 관계, 시술비용에 대한 부담, 치료 기간 등), 우울, 불안을 변수로 본 선행연구에서 ΔR^2^=.121–.377 및 R^2^=.209–.480으로 나타나[9,15] 이를 근거로 중간 효과크기(f^2^)인 .15를 기준으로 하였다. 예측변수는 15개, 검정력 (1–β)를 0.8로 두고 산출하였을 때 139명이었다. 탈락률 10%를 반영하여 총 150명에게 설문지를 배부하였고, 불성실한 응답자 10명을 제외하고 총 140명(응답률 93.3%)을 대상으로 조사하였다. 본 연구 대상자의 구체적인 선정기준은 다음과 같다.

첫째, 난임을 진단받고 의사에게 상담(자연주기법) 및 시술(배란 유도, 인공수정, 체외수정)을 받는 여성

둘째, 지남력의 장애가 없으며 글을 읽고 이해할 수 있는 여성

셋째, 다른 정신과적 질환이 없는 여성

넷째, 연구의 목적을 이해하고 참여하기를 동의한 여성

### 연구 도구

#### 일반적 특성

난임 여성과 배우자의 나이, 직업 유무, 결혼 기간, 종교, 현재 치료 방법, 난임 치료 기간, 가구의 평균 월 수입, 난임 치료에 임하는 대상자의 태도와 배우자의 태도, 아이의 중요성 정도, 임신에 대한 부담, 아이를 가장 바라는 사람, 임신에 부담을 가장 많이 주는 사람, 임신에 가장 협조적인 사람 등으로 이루어졌다. 난임 치료에 임하는 태도는 선행연구에 근거하여[16] 적극적(1점), 보통(2점), 소극적(3점)으로 분류하였고, 아이의 중요성 정도 또한 선행연구를 바탕으로[15] 보통(1점), 약간 중요함(2점), 중요함(3점), 아주 중요함(4점)으로 나누어 분류하였다.

#### 피로

본 연구에서는 Krupp 등[17]이 피로 측정을 위해 개발한 Fatigue Severity Scale (FSS)를 Chung과 Song [18]이 번안한 도구를 번안자로부터 사용 허가를 받아 사용하였다. FSS는 총 9문항으로 구성되어 있으며 ‘전혀 그렇지 않다' 1점부터 ‘매우 그렇다' 7점까지의 7점 척도이다. 총점은 문항별 점수의 평균으로 계산하며 1점에서 7점까지의 범위로, 평균값이 높을수록 피로가 심한 것을 의미한다. 평균 점수가 중앙값 4점 이상인 경우 피로군으로 해석하고 4점 미만인 경우 비피로군으로 분류한다. 선행연구에서 타당도가 확인되었고 Cronbach's alpha는 .94였다[18]. 본 연구에서 Cronbach's alpha는 .93이었다.

#### 우울

본 연구에서는 우울 측정을 위해 Depression Anxiety Stress Scale-21 (DASS-21) 도구[19] 중 우울 관련 7문항만을 사용하였다. DASS-21은 Henry와 Crawford [17]가 개발한 우울, 불안, 스트레스 척도(Depression Anxiety Stress Scale)로 각 증상을 측정하는 문항이 7문항씩, 총 21문항으로 이루어졌다. 우울 점수의 범위는 0–3점으로 구성되었으며 ‘전혀 해당되지 않음' 0점에서 ‘약간 또는 가끔 해당됨' 1점, ‘상당히 또는 자주 해당됨' 2점, ‘매우 많이 또는 거의 대부분 해당됨' 3점의 4점 Likert 척도로 구성되어 있다. 0점에서 21점까지로 점수가 높을수록 우울함의 정도가 심함을 의미한다. 우울의 정도는 정상(0–4), 약간 우울(5–6), 중간 정도 우울(7–10), 심한 우울(11–13), 극심한 우울(≥14)로 구분한다. 개발 당시 우울 영역의 Cronbach's alpha는 .88이었고[19], 본 연구에서 Cronbach's alpha는 .87이었다. 본 연구는 연구용으로 개방된 한국어판 DASS-21 도구를 사용하였다.

#### 부부 친밀도

본 연구에서 부부 친밀도의 측정은 Lee [13]가 개발한 도구를 도구 개발자의 동의를 받고 사용하였다. 본 도구는 총 15개 문항으로, 하위 영역으로 인지적 영역 5문항, 정서적 영역 5문항, 성적 영역 5문항으로 이루어졌다. 각 문항은 5점 Likert 척도로 점수의 범위는 ‘전혀 그렇지 않다' 1점에서부터 ‘매우 그렇다' 5점으로 구성되었다. 부부 친밀도 점수는 최저점 15점에서부터 최고점 75점까지로 총점이 높을수록 부부 친밀도가 높음을 의미한다. 본 도구 개발 당시 Cronbach's alpha는 .90이었고, 친밀감의 인지적 영역의 Cronbach's alpha는 .79, 정서적 영역의 Cronbach's alpha는 .77, 성적 영역의 Cronbach's alpha는 .83이다[13]. 본 연구에서 Cronbach's alpha는 .84이다.

#### 난임 관련 삶의 질

본 연구에서는 난임 여성의 삶의 질을 측정하기 위해 난임 관련 삶의 질 도구(The Fertility Quality of Life tool, FertiQoL)를 사용하였다[20]. 본 도구는 Boivin과 Schmidt [20]가 유럽생식배아협회(European Society of Human Reproduction and Embryology), 미국생식의학회(American Society for Reproductive Medicine)와 함께 제시한, 난임 문제가 있는 사람들의 삶의 질을 측정하기 위해 개발된 도구이다. 45개 언어로 번역된 도구 중 한국어판 도구를 사용하였으며, 국내 난임 여성을 대상으로 하여 타당도가 확인되었다[11]. 총 34문항의 도구로 핵심 삶의 질(core FertiQoL) 24문항과 치료 삶의 질(treatment FertiQoL) 10문항, 전반적인 건강상태 평가(overall physical health) 1문항, 삶의 질 만족도(satisfaction) 1문항으로 구성된다. 핵심 삶의 질은 정서적(emotional) 영역, 심신(mind-body) 영역, 관계적(relational) 영역, 사회적(social) 영역으로 각각 6문항씩 이루어졌고, 치료 삶의 질은 치료환경(treatment environment)을 묻는 6문항, 치료환경을 참을 수 있는 인내심(treatment tolerability)을 묻는 2문항으로 되어 있으며, 각 문항은 0점에서 4점까지로 되어 있다. 총 삶의 질(total FertiQoL)은 핵심 삶의 질과 치료 삶의 질 영역의 평균 점수로 나타내며, 전반적인 건강상태 평가(1문항)와 삶의 질 만족도(1문항) 점수는 FertiQoL 점수에는 사용되지 않는다. 모든 영역은 0점에서 100점으로 환산하여 평가하며, 총 점수가 높을수록 난임 관련 삶의 질이 높음을 의미한다. 본 도구는 개발 당시 Cronbach's alpha는 .92였고[20], 본 연구에서 Cronbach's alpha는 .91이었다. 본 도구는 연구용으로 개방된 도구이다.

### 자료 수집

자료수집은 2018년 2월 1일부터 4월 30일까지 진행됐으며, 해당 난임 전문병원의 간호 부서장 및 진료과 의사에게 허락을 받은 후 연구에 대한 대상자 모집에 관한 홍보물과 설명문을 병원에 전달하였다. 설문 조사는 대상자가 진료를 위해 내원 시 대상자 모집 홍보를 한 후 대상자가 동의를 한 경우에 진료를 대기하는 동안이나 진료를 마친 후에 대상자에게 시행하였다. 설문지는 자가 보고형 설문지를 이용하여 대상자가 직접 작성하였으며 편안하고 조용한 곳에서 조사되었고, 응답하는 데 약 20분 정도 소요되었다. 설문지는 현장에서 직접 회수하였다. 대상자에게는 감사의 표시로 기념품을 제공하였다.

### 자료 분석 방법

본 연구의 자료 분석을 위해 IBM SPSS Statistics for Windows, ver. 25.0 프로그램(IBM Corp., Armonk, NY, USA)을 이용하였다. 통계적 유의성은 *p*<.05로 하였다.

1) 일반적 특성은 실수와 백분율, 평균과 표준편차, 범위, 최대값, 최소값, 왜도와 첨도를 이용하여 분석하였다.

2) 난임 여성의 피로, 우울, 부부 친밀도, 난임 관련 삶의 질은 기술통계를 이용하였다.

3) 본 연구에 사용된 도구의 신뢰도는 Cronbach's alpha coefficient를 구해 검증하였다.

4) 일반적 특성에 따른 피로, 우울, 부부 친밀도, 난임 관련 삶의 질의 정도의 차이는 independent t-test 및 ANOVA, 사후분석은 Scheffé test, 변수의 정규성 검정은 Kolmogorov–Smirnov검정을 이용하여 분석하였다.

5) 난임 여성의 주요변수의 관계는 Pearson's correlation coefficient로 분석하였다.

6) 난임 여성의 난임 관련 삶의 질 영향요인을 알아보기 위해 stepwise multiple regression을 실시하였다.

### 수집된 자료 관리

수집된 자료는 설문을 마침과 동시에 봉투에 밀봉하였고, 수집된 자료는 연구자만 관리하며 지정된 장소에 보관하고 접근 가능자는 연구자로 제한하였고 전자파일은 암호를 걸어 보관하였다. 수집된 자료는 보고서 완료 3년이 지난 시점에서 분쇄기를 이용하여 안전하게 폐기할 것이며, 연구용 컴퓨터에 저장된 파일 및 이동식 디스크에 저장된 자료들은 영구 삭제 예정이다.

## Results

### 대상자의 일반적 특성

대상자의 연령은 평균 35.61±4.29세로 35세 이상이 58.6% (82명)였고, 배우자의 연령은 평균 37.81±4.93세이며 35세 이상이 69.3% (97명)로 나타났다. 직업을 갖고 있는 대상자는 75.0% (105명)였고, 대상자들의 결혼 기간은 평균 48.46±40.23개월로 나타났으며, 종교를 갖고 있는 대상자는 64.3% (90명)으로 나타났다. 현재 난임 시술은 체외수정 28.6% (40명), 배란 유도 27.9% (39명), 인공수정 23.6% (33명) 순이었고, 난임 치료 기간은 평균 15.91±21.74개월로 나타났으며, 월평균 소득은 400만 원에서 500만 원 미만이 35.7% (50명)로 가장 많았다. 난임 치료에 임하는 태도는 3점 만점에 평균 1.56±0.57점으로, 적극적인 태도 47.1% (66명), 보통 49.3% (69명), 소극적인 태도 3.6% (5명)로 나타났다. 난임 치료에 임하는 배우자의 태도는 3점 만점에 평균 1.60±0.63점으로 적극적인 태도 47.1% (66명), 보통 44.3% (62명), 소극적인 태도 7.9% (11명)로 나타났다. 아이의 중요성 정도는 4점 만점에 평균 3.56±1.09점으로 아이를 갖는 것이 ‘중요하다' 40.0% (56명)가 가장 많았고, 임신에 대한 부담을 묻는 문항은 ‘부담스럽다'가 87.1% (122명)로 나타났다. 아이를 가장 많이 기대하는 사람은 난임 여성 본인이 40.0% (56명), 부모님 33.6% (47명) 순서로 나타났고, 임신에 부담을 주는 사람에는 부모님 25.7% (36명)이 가장 높았으며 임신에 가장 협조적인 사람은 배우자 72.9% (102명)로 나타났다(Table 1).

### 대상자의 피로, 우울, 부부 친밀도와 난임 관련 삶의 질

본 연구에서 피로의 정도는 평균 평점 3.60±1.33점으로 피로가 있는 여성이 37.1%였다. 우울의 정도는 평균 3.74±3.93점이었고, 우울이 있는 여성이 31.4%였다. 부부 친밀도는 평균 51.16±7.40점으로 나타났다. 총 난임 관련 삶의 질 점수는 평균 67.49±12.05점으로 나타났고, 도구의 하부 영역으로 일반적인 건강상태는 평균 2.47±0.75점, 일반적인 삶의 만족은 평균 2.74±0.70점이었다. 하부 영역인 정서적 영역의 평균 점수는 68.84±17.28점, 심신 영역은 평균 69.70±18.66점, 관계적 영역은 평균 70.51±15.21점, 사회적 영역은 평균 67.11±15.06점이었고, 치료적 환경 영역은 평균 62.50±13.57점, 치료적 인내심은 평균 66.29±14.74점으로 나타났다(Table 2). 본 연구 변수(피로, 우울, 부부 친밀도와 삶의 질)의 왜도와 첨도는 ±1에 분포하고 있으므로 정규분포의 가정에서 크게 벗어나지 않는 것으로 나타났으며 변수의 정규성 검정은 Kolmogorov–Smirnov 검정을 이용하였고 *p*>.05로 정규분포를 보였다.

### 대상자의 일반적 특성에 따른 피로, 우울, 부부 친밀도와 난임 관련 삶의 질

대상자의 피로는 일반적 특성에 따라 차이가 없었다. 우울은 난임 치료에 임하는 배우자의 태도(F=5.22, *p*=.007)에 따라 유의한 차이가 있었고 사후분석 결과 배우자의 태도가 수동적인 군이 적극적이고 보통인 경우보다 우울이 더 높았다. 부부 친밀도는 배우자의 태도가 적극적인 군이 보통과 수동적인 군보다 더 높게 나타났고(F=7.50, *p*=.001) 임신에 부담을 가장 많이 주는 사람에 따라 차이가 있었다(F=2.82, *p*=.028). 또한 임신에 가장 협조적인 사람에 따라 차이가 있었고(F=3.81, *p*=.006) 사후분석 결과 임신에 가장 협조적인 사람이 남편인 경우가 협조적인 사람이 없는 경우보다 부부 친밀도가 높게 나타났다. 난임 관련 삶의 질 정도는 대상자의 일반적인 특성 중 난임 치료에 임하는 대상자 태도(F=3.45, *p*=.035)와 난임 치료에 임하는 배우자의 태도(F=5.06, *p*=.008), 임신에 부담을 가장 많이 주는 사람(F=4.18, *p*=.003)에 따라 유의한 차이가 있었다. 사후검정 결과 난임 치료에 임하는 대상자의 태도가 적극적인 군은 보통인 군보다 난임 관련 삶의 질 점수가 높았고, 배우자의 태도가 적극적인 군은 배우자의 태도가 수동적인 군보다 난임 관련 삶의 질 점수가 더 높았다. 임신에 부담을 주는 사람이 없는 군은 임신에 부담을 주는 사람이 부모님인 군보다 난임 관련 삶의 질이 더 높게 나타났다(Table 3).

### 대상자의 피로, 우울, 부부 친밀도와 난임 관련 삶의 질 간의 관계

난임 여성의 피로, 우울과 난임 관련 삶의 질은 음의 상관관계를 나타냈고(r=–.42, *p*<.001; r=–.56, *p*<.001), 부부 친밀도와 난임 관련 삶의 질은 양의 상관관계를 나타냈다(r=.30, *p*<.001). 또한, 피로와 우울은 양의 상관관계를 나타냈고(r=.33, *p*<.001), 피로와 부부 친밀도는 음의 상관관계(r=–.19, *p*=.025), 우울과 부부 친밀도는 음의 상관관계(r=–.38, *p*<.001)를 나타냈다(Table 4).

### 난임 관련 삶의 질 영향요인

난임 관련 삶의 질 영향요인을 규명하기 위하여 피로, 우울, 부부 친밀도와 일반적 특성 중 난임 관련 삶의 질에 유의한 차이가 있었던 난임 치료에 임하는 대상자의 태도, 배우자의 태도와 임신에 부담을 가장 많이 주는 사람을 더미 변수로 처리하여 단계적 회귀분석을 실시하였다. 단계적 다중회귀분석 결과 부부 친밀도, 난임 치료에 임하는 대상자의 태도와 임신에 부담을 주는 사람의 더미 변수는 모두 회귀식에서 제외되었고, 우울(β=–.44, *p*<.001), 피로(β=–.27, *p*<.001), 배우자의 태도(β=–.19, *p*=.007) 순으로 난임 관련 삶의 질에 유의한 영향을 주는 것으로 나타났다. 본 연구 결과, 우울 점수가 낮을수록, 피로 점수가 낮을수록, 난임 치료에 임하는 배우자의 태도가 적극적일수록 난임 관련 삶의 질이 높아졌다. 난임 관련 삶의 질을 설명하는 전체 설명력은 40.5%였다. Cook's distance 통계량을 이용하여 영향력을 분석한 결과 1.0 이상을 보이는 값은 없었고, 분산팽창지수(variance inflation factor)는 1.00-1.35로 기준치 10 이하로 나타나 독립변수간의 다중공선성 문제는 없었다. 수행한 회귀분석 모형이 적합한지에 대한 모형 적합도 검정을 위해 표준화된 잔차에 대한 Kolmogorov–Smirnov 정규성 검정(Z=.895, *p*=.400)과, 잔차 등분산성 Breusch–Pagan 검정(χ2=7.06, *p*=.316)을 실시하였다. 잔차 분석 결과 표준화된 잔차의 정규성과 등분산성이 만족되어 회귀모형이 적합하였다(Table 5).

## Discussion

본 연구는 난임 여성의 난임 관련 삶의 질 영향요인을 파악하여 난임 여성의 삶의 질을 높이기 위한 간호 중재 개발의 기초자료를 제공하고자 시도되었다. 난임 여성은 난임 치료 과정 중 검사와 처치 등으로 인해 다양한 신체적, 심리적, 관계적 문제를 경험하므로 난임 여성의 삶의 질에 의료진의 더욱 많은 관심이 필요하다. 본 연구를 통해 본 연구에서 난임 관련 삶의 질 영향요인으로 일반적 특성, 피로, 우울과 부부 친밀도를 확인한 결과 우울, 피로와 배우자의 태도 순으로 영향을 미치는 것으로 확인되었다.

본 연구 결과, 난임 여성의 난임 관련 삶의 질 점수는 100점 만점으로 환산하였을 때 67.49점으로, 동일한 도구를 사용한 선행연구와 비교하였을 때 국내 난임 여성 203명 대상의 연구[21]에서 보고한 69.5점 및 180명의 이란 난임 여성을 대상으로 한 연구[22]의 67.36점과 유사하며, 중국의 난임 여성 498명에게서 측정한[23] 64.54점보다는 높은 점수이다. 이러한 결과는 대상자의 차이로 보이는데, Li 등[23]의 연구는 체외수정을 하고 있는 여성을 대상으로 한 반면, 본 연구와 Maroufizadeh 등[22]의 연구 대상자는 난임 진단을 받은 여성으로 치료방법에 대한 구분 없이 대상자를 선정하여 난임 관련 삶의 질이 상대적으로 높았을 것으로 생각된다. 본 연구에서 난임 관련 삶의 질은 난임 치료에 임하는 여성의 태도와 배우자의 태도, 임신에 가장 부담을 주는 사람에 따라 차이가 있었다. 이것은 난임 치료에 대한 부담감과 배우자와의 관계에 따라 난임 관련 삶의 질에 차이가 난다는 Kim과 Shin [21]의 연구결과와 유사하며, 난임 여성의 삶의 질은 배우자와 주변 사람들의 임신에 대한 기대 등에 영향을 받았음을 알 수 있다. 이것은 추후 난임 여성의 건강 관리를 위한 프로그램 계획 시 배우자와 가족을 포함시키는 것이 필요함을 보여준다.

난임 여성의 난임 관련 삶의 질에 가장 큰 영향을 미치는 요인은 우울로 나타났다. 본 연구에서 대상자의 31.4%는 우울한 증상이 있는 것으로 나타났는데, 이러한 결과는 Beck의 우울 측정도구를 사용한 Hwang [24]의 연구에서 난임 여성 중 49.4%는 우울한 증상을 느끼고 있다고 보고한 것과 유사하다. 반면, Center for Epidemiologic Studies Depression Scale (CES-D) 도구를 활용하여 측정했을 때 94%의 우울 증상이 있었던 Kim 등[10]의 연구와는 차이가 있었는데 이러한 결과는 난임 여성인 대상자들이 보조생식술의 단계를 겪고 있는 당시 치료 상황에 따른 영향으로 파악된다. 즉, 본 연구 결과와 우울의 정도가 유사했던 Hwang [24]의 연구는 난임 치료의 모든 과정 단계를 포함하고 있으나, Kim 등[10]은 보조생식술을 받는 여성만을 대상으로 하여 이에 따른 차이가 있다고 생각된다. 또한, 난임 여성의 우울은 난임 치료에 임하는 배우자의 태도가 소극적일 때 더 심한 것으로 나타났다. 이는 난임 여성의 우울이 배우자의 지지나 배우자와의 관계적 측면과 관련이 있음을 나타내는 결과로, 난임 여성의 우울 감소를 위해서는 배우자의 역할이 중요함을 제시하고 있다. 난임 관련 삶의 질에 대한 자기/상대방 효과에 대한 연구에서[21] 본인의 우울뿐만 아니라 배우자의 우울이 난임 관련 삶의 질에 영향을 미친다는 연구 결과는, 난임 여성을 돌보는 간호사는 난임 여성뿐 아니라 배우자에게도 관심을 갖고 배우자의 태도와 우울 등을 사정할 필요가 있음을 나타낸다.

본 연구 결과, 난임 여성의 난임 관련 삶의 질에 영향을 미치는 두 번째 요인은 피로로 나타났고 피로군으로 분류되는 대상자는 37%에 이르렀다. 본 연구의 대상자들은 보조생식술 단계가 52.2%로 반복적인 진료와 검사 등이 신체적 부담과 긴장을 유발하였을 것이고, 대상자의 75%가 직업이 있는 여성이라는 점 또한 피로를 증가시켰을 것으로 볼 수 있다. 피로는 나이가 들수록 증가한다고 하였는데[25] 본 연구에 참여한 대상자와 배우자의 평균 연령이 35세 이상으로 신체적, 생리적 기능이 고령화되어 있어 감소된 생식 능력과 신체 능력이 대상자의 피로에 영향을 미칠 수 있었을 것으로 보인다. 또한 본 연구에서 피로는 우울과도 상관관계가 있는 것으로 나타났는데, 피로가 우울의 잔여 증상(residual symptom)이라고 한 것처럼[26] 난임 여성의 피로와 우울에 대해 정확히 사정하고 이러한 문제를 경험하는 대상자를 위한 피로와 우울 감소 중재 프로그램을 개발하는 등 난임 관련 삶의 질 향상을 위한 의료진의 관심이 필요하다. 그러나, 난임 여성의 피로와 관련된 연구는 많이 시도되지 않은 실정으로 난임 여성의 피로 및 관련 요인에 대한 추가적인 탐색 및 우울, 피로와 난임 관련 삶의 질에 대한 매개 효과 혹은 조절 효과 등에 대한 연구 시행이 필요하다.

본 연구에서 난임 관련 삶의 질에 영향을 미친 세 번째 요인은 배우자의 태도로 확인되었다. 난임 관련 삶의 질은 배우자의 태도가 적극적인 경우가 소극적일 때 더 높게 나타났다. 난임 치료에 임하는 배우자의 태도가 적극적인 경우에 부부 친밀도가 높았던 것을 통해 부부 관계에서 배우자가 가장 중요한 사람이라는 것을 알 수 있었고, 임신에 부담을 가장 많이 주는 사람이 있는 경우와 임신에 가장 협조하는 사람을 확인함으로써 배우자의 적극적인 태도, 배우자의 협조와 지지가 난임 여성에게 중요함을 확인하였다. 이것은 유럽의 난임 부부 540명을 대상으로 한 연구에서[27] 부부의 적극적인 태도가 중립적인 태도보다 난임 관련 삶의 질이 높다고 보고한 것과 맥락을 같이 한다. 즉, 치료에 대한 임부와 배우자의 적극적인 태도가 중요하며 이는 배우자가 난임 여성에게 부담을 주기보다 심리적·정서적인 지지체계가 됨으로써 난임을 여성만의 문제가 아닌 부부가 함께 겪고 있는 공동의 문제로 인식할 수 있게 되는 의미가 있다고 볼 수 있다. 이렇듯 난임 여성에게 배우자의 태도는 난임 관련 삶의 질에 영향을 미치는 주요 변수이므로 배우자의 태도 관리를 통해 난임 여성뿐 아니라 부부의 건강 증진에 기여할 수 있을 것이다.

본 연구에서는 부부 친밀도가 난임 관련 삶의 질에 영향을 미치는 요인으로는 확인되지 않았으나 부부 친밀도가 높을수록 난임 관련 삶의 질이 높은 것으로 나타났다. 이탈리아에서 보조생식술을 시작하는 589명의 환자(여성 301명, 남성 288명)를 대상으로 난임 관련 삶의 질과 부부 관계에 관해 조사한 연구에서[28] 부부 관계와 난임 관련 삶의 질의 관계적 점수는 여성과 남성에서 크게 차이가 없었으나 부부 관계가 긍정적일 때 난임 여성들의 전반적인 삶의 질이 향상된다고 보고하여, 난임 부부의 친밀도를 향상시킬 수 있는 방안에 대한 고려가 필요하다. 부부 친밀도는 치료에 임하는 배우자의 태도가 적극적이고 임신에 가장 협조적인 사람이 배우자일 경우에 높게 나타났고 임신에 부담을 많이 주는 사람이 없을 경우에 높게 나타났다. 이는 배우자의 태도가 적극적일 경우 결혼 만족도가 높다는 선행연구[29]와 맥락을 같이 한다. 즉, 무엇보다도 배우자의 적극적인 태도와 협조가 난임 여성의 부부 친밀도를 높이며, 이는 삶의 질 향상으로 이어질 수 있음을 확인하였다. 따라서 추후 배우자의 태도를 객관적으로 측정할 수 있는 도구를 활용하여 부부 친밀도와 난임 관련 삶의 질에 대한 인과관계를 확인하는 후속 연구가 필요하다고 생각된다.

본 연구는 난임 치료 단계 및 치료 기간의 분류 없이 횡단적으로 확인하였고, 시술 횟수와 유산력 등의 요소를 확인하지 않았다는 제한점이 있다. 추후 연구에서 이에 대한 고려를 포함하여 난임 관련 삶의 질 영향요인을 확인하는 연구가 필요할 것으로 보인다. 또한, 본 연구에서 사용한 난임 관련 삶의 질 도구에 우울 문항이 포함되어 있어 우울의 설명력이 높게 나왔을 가능성이 있으므로 이에 대한 고려가 필요하다. 추가적으로, 한 지역에서 시행되어 일반화하기에 어려움이 있다. 그럼에도 불구하고 본 연구는 난임 관련 삶의 질 영향요인에 대해 인구학적 특성을 비롯한 신체적, 심리적, 관계적 측면에서 주요한 원인으로 구분하고 난임 여성의 포괄적인 부분을 고려하여 삶의 질을 파악했다는 것에 의미가 있다고 볼 수 있다. 즉, 난임 여성이 주로 경험하는 우울뿐 아니라 난임 과정 중 경험하는 피로에도 관심을 가져야 함을 확인하였고, 특히 치료에 임하는 배우자의 태도가 난임 관련 삶의 질에 주요한 영향을 미침을 확인하였다. 따라서 난임 여성 대상의 피로 및 우울과 배우자의 태도에 대한 사정에 비중을 두어 이러한 문제가 있는 대상자들을 위한 중재 프로그램 개발 시 피로 감소, 우울 감소 및 배우자의 치료에 대한 인식과 자세를 긍정적이고 적극적으로 고취할 수 있는 내용을 포함하여 개발하고 이를 적용하는 것이 필요함을 확인하였다.

## Conclusion

본 연구는 난임 여성의 난임 관련 삶의 질 영향요인을 확인하고 삶의 질을 향상시키기 위한 효과적인 간호중재 프로그램 개발에 필요한 기초자료를 제공하기 위해 시행되었다. 난임 관련 삶의 질 영향요인은 우울, 피로, 난임 치료에 임하는 배우자의 태도로 확인되었다. 이로써 난임 여성의 삶의 질을 향상시키기 위해서는 우울과 피로를 사정하여 중재하고 난임 치료에 임하는 배우자의 태도를 관리하는 것이 필요함을 알 수 있다.

이상의 결과를 통하여 다음과 같은 제언을 하고자 한다. 난임 여성의 삶의 질에 영향을 미치는 요인을 중심으로 간호중재 프로그램을 개발 및 적용하여 효과를 검증하는 후속 연구가 필요하다. 종단적 연구를 통해 난임 여성을 대상으로 삶의 질에 영향을 미치는 요인을 확인하는 후속 연구 및 난임 치료 단계별로 신체적, 심리적, 관계적 요인이 난임 관련 삶의 질에 미치는 영향을 파악하는 연구를 제언한다.

## Figures and Tables

**Table 1.** General characteristics of subjects (N=140)

Variable	Categories	n (%) or Mean ± SD
Women’s age (year)		35.61 ± 4.29
	< 30	9 (6.4)
	30–34	49 (35.0)
	≥ 35	82 (58.6)
Husband’s age (year)		37.81 ± 4.93
	< 30	4 (2.9)
	30–34	39 (27.9)
	≥ 35	97 (69.3)
Employment status	Employed	105 (75.0)
	Unemployed	35 (25.0)
Marital duration (month)		48.46 ± 40.23
Religion	Yes	90 (64.3)
	No	50 (35.7)
Treatment	Ovulation induction	39 (27.9)
	IUI	33 (23.6)
	IVF	40 (28.6)
	Others^†^	28 (20.0)
Duration of infertility treatment (month)		15.91 ± 21.74
Monthly income (× 10,000, KRW)	< 200	14 (10.0)
	200–299	20 (14.3)
	300–399	20 (14.3)
	400–499	50 (35.7)
	≥ 500	36 (25.7)
Attitude toward infertility treatment	Women (range, 1–3)	1.56 ± 0.57
	Active	66 (47.1)
	Medium	69 (49.3)
	Passive	5 (3.6)
	Husband (range, 1–3)	1.60 ± 0.63
	Active	66 (47.1)
	Medium	62 (44.3)
	Passive	11 (7.9)
	Missing value	1 (0.7)
Importance of having a child (range, 1–4)		3.56 ± 1.09
	Neutral	22 (15.7)
	Slightly important	20 (14.3)
	Important	56 (40.0)
	Strongly important	41 (29.3)
	Missing value	1 (0.7)
Burden on pregnancy	Yes	122 (87.1)
	No	18 (12.9)
Person most hoping for a child	Women	56 (40.0)
	Husband	31 (22.1)
	Parents	47 (33.6)
	Others	3 (2.1)
	None	3 (2.1)
Most pressuring person regarding pregnancy	Women	18 (12.9)
	Husband	19 (13.6)
	Parents	36 (25.7)
	Others	7 (5.0)
	None	60 (42.9)
Most supportive person for pregnancy	Women	19 (13.6)
	Husband	102 (72.9)
	Parents	7 (5.0)
	Others	7 (5.0)
	None	5 (3.6)

IUI: Intrauterine insemination; IVF: in vitro fertilization; KRW: Korean won.

† Counseling about natural pregnancy.

**Table 2.** Fatigue, depression, marital intimacy, and fertility-related quality of life (N=140)

Variable	Categories	n (%) or Mean ± SD
Fatigue		3.60 ± 1.33
	Yes (≥ 4)	52 (37.1)
	No (< 4)	88 (62.9)
Depression		3.74 ± 3.93
	Normal (0–4)	96 (68.6)
	Mild (5–6)	15 (10.7)
	Moderate (7–10)	19 (13.6)
	Severe (11–13)	5 (3.6)
	Extremely severe (≥ 14)	5 (3.6)
Marital intimacy		51.16 ± 7.40
	Perceptional part	18.31 ± 2.94
	Emotional part	16.77 ± 3.00
	Sexual part	12.85 ± 2.13
Quality of life		67.49 ± 12.05
	Overall physical health	2.47±0.75
	Quality of life satisfaction	2.74±0.70
	Core FertiQoL	
	Emotional subscale	68.84 ± 17.28
	Mind-body subscale	69.70 ± 18.66
	Relational subscale	70.51 ± 15.21
	Social subscale	67.11 ± 15.06
	Treatment FertiQoL	
	Environment subscale	62.50 ± 13.57
	Tolerability subscale	66.29 ± 14.74

FertiQoL: Fertility quality of life tool.

**Table 3.** Comparison of fatigue, depression, marital intimacy, and fertility-related quality of life according to subjects’ characteristics (N=140)

Variable	Categories	n (%)	Fatigue		Depression		Marital intimacy		Fertility-related quality of life	
			Mean ± SD	t/F (*p*)	Mean ± SD	t/F (*p*)	Mean ± SD	t/F (*p*)	Mean ± SD	t/F (*p*)
Women’s age (year)	< 30	9 (6.4)	29.33 ± 10.02	0.39 (.681)	2.44 ± 2.30	1.47 (.234)	49.33 ± 4.90	1.86 (.159)	71.41 ± 9.99	0.70 (.500)
	30–34	49 (35.0)	33.12 ± 12.01		3.22 ± 3.68		52.76 ± 6.59		66.38 ± 11.26	
	≥ 35	82 (58.6)	32.30 ± 12.16		4.20 ± 4.18		50.40 ± 7.97		67.73 ± 12.72	
Husband’s age (year)	< 30	4 (2.9)	34.75 ± 6.50	0.11 (.899)	7.50 ± 4.12	2.99 (.054)	54.00 ± 2.94	0.48 (.623)	61.28 ± 9.43	0.56 (.571)
	30–34	39 (27.9)	32.72 ± 12.08		2.87 ± 3.00		51.67 ± 6.86		68.00 ± 12.26	
	≥ 35	97 (69.3)	32.18 ± 12.13		3.94 ± 4.17		50.84 ± 7.74		67.54 ± 12.10	
Job	Yes	105 (75.0)	33.40 ± 12.17	1.73 (.270)	3.57 ± 4.02	–.89 (.912)	50.90 ± 7.79	–.72 (.134)	67.38 ± 12.39	0.04 (.846)
	No	35 (25.0)	29.40 ± 10.85		4.26 ± 3.67		51.94 ± 6.12		67.84 ± 11.13	
Duration of infertility treatment (month)	< 49	94 (67.1)	33.37 ± 11.48	1.38 (.454)	3.33 ± 3.63	–1.79 (.244)	51.45 ± 6.65	0.66 (.058)	67.40 ± 12.02	0.02 (.895)
	≥ 49	46 (32.9)	30.41 ± 12.73		4.59 ± 4.40		50.57 ± 8.80		67.69 ± 12.25	
Religion	Yes	90 (64.3)	31.76 ± 11.98	0.73 (.394)	3.51 ± 4.09	0.88 (.351)	51.00 ± 7.45	.011 (.737)	68.55 ± 11.99	1.94 (.166)
	No	50 (35.7)	33.56 ± 11.91		4.16 ± 3.63		51.44 ± 7.39		65.60 ± 12.05	
Current state of infertility treatment	Ovulation induction	39 (27.9)	32.51 ± 12.35	0.15 (.927)	2.85 ± 3.27	1.19 (0.317)	51.31 ± 6.75	0.38 (.769)	68.45 ± 11.38	0.48 (.697)
	IUI	33 (23.6)	31.33 ± 10.31		4.33 ± 4.34		50.64 ± 6.79		65.41 ± 13.51	
	IVF	40 (28.6)	33.25 ± 11.20		3.68 ± 3.67		52.05 ± 7.98		68.39 ± 11.31	
	Other	28 (20.0)	32.29 ± 14.48		4.39 ± 4.55		50.29 ± 8.31		67.32 ± 12.51	
Monthly income (× 10,000, KRW)	< 200	14 (10.0)	32.86 ± 10.18	0.01 (1.000)	5.64 ± 4.78	1.66 (.164)	47.00 ± 7.86	1.79 (.135)	64.14 ± 11.37	1.07 (.374)
	200–299	20 (14.3)	32.45 ± 12.87		4.45 ± 4.15		49.45 ± 6.04		65.64 ± 12.29	
	300–399	20 (14.3)	32.20 ± 12.19		4.35 ± 4.56		52.00 ± 7.51		64.93 ± 14.28	
	400–499	50 (35.7)	32.52 ± 12.41		3.10 ± 2.98		51.90 ± 6.59		68.42 ± 10.29	
	≥ 500	36 (25.7)	32.14 ± 11.90		3.17 ± 4.12		52.22 ± 8.51		69.97 ± 13.04	
Women’s attitude	Active^a^	66 (47.1)	33.42 ± 12.36	0.46 (.632)	3.58 ± 3.95	0.13 (.882)	52.47 ± 7.58	2.02 (.136)	70.00 ± 12.19	3.45 (.035)
	Medium^b^	69 (49.3)	31.45 ± 11.27		3.91 ± 3.97		49.93 ± 7.24		64.83 ± 11.69	a> b^†^
	Passive^c^	5 (3.6)	32.00 ± 16.73		3.60 ± 3.78		50.80 ± 5.07		71.11 ± 12.05	
Husband’s attitude	Active^a^	66 (47.1)	33.82 ± 11.90	2.22 (.113)	3.27 ± 3.45	5.22 (.007)	53.26 ± 7.25	7.50 (.001)	70.31 ± 11.53	5.06 (.008)
	Medium^b^	62 (44.3)	29.98 ± 11.60		3.60 ± 3.94	c > a,b^†^	50.18 ± 6.25	a,b > c^†^	66.26 ± 11.65	a> c^†^
	Passive^c^	11 (7.9)	35.73 ± 11.85		7.27 ± 5.24		45.18 ± 9.63		59.22 ± 12.40	
Importance of having a child (n=139)	Neutral	22 (15.7)	33.27 ± 11.53	0.11 (.954)	4.05 ± 4.55	0.23 (.874)	51.18 ± 8.40	0.18 (.908)	66.11 ± 9.34	0.42 (.736)
	Slightly important	20 (14.3)	31.25 ± 12.93		3.35 ± 3.38		50.65 ± 5.16		68.44 ± 11.60	
	Important	56 (40.0)	32.50 ± 12.08		3.91 ± 4.08		50.84 ± 7.97		66.56 ± 13.22	
	Strongly important	41 (29.3)	32.02 ± 11.83		3.41 ± 3.68		51.85 ± 7.25		68.89 ± 12.20	
Burden on pregnancy	Yes	122 (87.1)	32.44 ± 11.68	0.11 (.245)	3.74 ± 3.75	–0.04 (.213)	51.09 ± 7.58	–0.28 (.316)	66.87 ± 12.15	2.55 (.113)
	No	18 (12.9)	32.11 ± 13.96		3.78 ± 5.13		51.61 ± 6.23		71.70 ± 10.72	
Person most hoping for a child	Women	56 (40.0)	30.36 ± 11.09	0.71 (.584)	3.55 ± 4.32	0.63 (.641)	52.23 ± 7.94	1.15 (.336)	68.09 ± 12.03	0.21 (.934)
	Husband	31 (22.1)	33.48 ± 12.84		4.03 ± 3.26		50.71 ± 7.47		67.20 ± 9.70	
	Parents	47 (33.6)	33.96 ± 12.17		3.70 ± 4.00		50.70 ± 6.77		66.57 ± 13.62	
	None	3 (2.1)	32.00 ± 15.10		2.00 ± 2.00		43.67 ± 4.51		71.06 ± 10.12	
	Others	3 (2.1)	35.33 ± 14.64		6.67 ± 2.52		50.33 ± 6.51		70.25 ± 16.45	
Most pressuring person regarding pregnancy	Women^a^	18 (12.9)	29.89 ± 13.15	0.54 (.707)	3.33 ± 3.24	2.13 (.080)	53.72 ± 7.65	2.82 (.028)	68.44 ± 12.74	4.18 (.003)
	Husband^b^	19 (13.6)	35.00 ± 12.94		5.21 ± 3.98		48.05 ± 8.24		62.20 ± 11.69	e> c^†^
	Parents^c^	36 (25.7)	33.47 ± 10.89		4.56 ± 4.15		50.53 ± 6.73		63.94 ± 12.60	
	Others^d^	7 (5.0)	32.14 ± 14.68		4.71 ± 3.64		46.00 ± 5.32		62.20 ± 11.03	
	None^e^	60 (42.9)	31.72 ± 11.73		2.80 ± 3.85		52.35 ± 7.20		71.63 ± 10.46	
Most supportive person for pregnancy	Women^a^	19 (13.6)	34.53 ± 13.27	0.31 (.868)	4.05 ± 4.09	1.80 (.133)	50.63 ± 8.34	3.81 (.006)	66.12 ± 12.66	0.82 (.513)
	Husband^b^	102 (72.9)	32.39 ± 11.94		3.50 ± 3.89		52.25 ± 7.11	b> e^†^	68.24 ± 11.94	
	Parents^c^	7 (5.0)	29.29 ± 10.40		3.71 ± 3.30		47.29 ± 4.50		63.34 ± 12.02	
	Others^d^	5 (3.6)	31.29 ± 13.09		3.29 ± 2.06		47.00 ± 3.65		69.44 ± 13.11	
	None^e^	7 (5.0)	30.40 ± 9.81		8.20 ± 5.40		42.00 ± 8.46		60.56 ± 11.31	

IUI: Intrauterine insemination; IVF: in vitro fertilization; KRW: Korean won.

† Scheffé test.

**Table 4.** Correlations among fatigue, depression, marital intimacy, and fertility-related quality of life (N=140)

Variable	r (*p*)			
	Fatigue	Depression	Marital intimacy	Fertility-related quality of life
Depression	.33 (< .001)	1		
Marital intimacy	–.19 (.025)	–.38 (< .001)	1	
Fertility-related quality of llife	–.42 (< .001)	–.56 (< .001)	.30 (< .001)	1

**Table 5.** Factors influencing fertility-related quality of life (N=140)

Variable	Step 1					Step 2					Step 3					
	B	SE	β	t	*p*	B	SE	β	t	*p*	B	SE	β	t	*p*	ΔR^2^
(constant)	74.05	1.16		63.79	< .001	81.02	2.36		34.35	< .001	81.02	3.20		27.25	< .001	
Depression	–1.72	0.21	–.57	-8.03	< .001	–1.47	0.22	–.49	–6.72	< .001	–1.47	0.22	–.44	–6.04	< .001	.320
Fatigue						–0.25	0.07	–.24	–3.36	.001	–0.25	0.07	–.27	–3.79	< .001	.052
Husband’s attitude											–3.54	1.30	–.19	–2.73	.007	.033
	Adjusted R^2^=.315, F (*p*)=64.41 (<.001)					Adjusted R^2^=.372, F (*p*)=40.27 (<.001)					Adjusted R^2^=.405, F (*p*)=30.59 (<.001)					
